# Lower Limb Pain Attributed to Bone Marrow Edema Syndrome: A Commonly Ignored Pathology

**DOI:** 10.7759/cureus.7679

**Published:** 2020-04-15

**Authors:** Theodore Balfousias, Efthimios J Karadimas, Despoina D Kakagia, Alexandros Apostolopoulos, Athanasios Papanikolaou

**Affiliations:** 1 Orthopaedics, Korgialenio-Benakio Hellenic Red Cross Hospital, Athens, GRC; 2 Orthopaedics, General Hospital Hellenic Red Cross "Korgialenio-Benakio, Athens, GRC; 3 Plastic Surgery, Democritus University of Thrace, Alexandroupoli, GRC; 4 Orthopaedics, East Surrey Hospital/Surrey and Sussex Healthcare National Health Service Trust, Redhill, GBR; 5 Orthopaedics, General Hospital Hellenic Red Cross "Korgialenio-Benakio", Athens, GRC

**Keywords:** bone marrow edema, muskuloskeletal mri, transient osteoporosis of the hip, lower limb pain

## Abstract

Bone marrow edema syndrome (BMES) is a highly uncommon, self-limited syndrome of unclear etiology. The syndrome most commonly affects middle-aged men. Magnetic resonance imaging is essential for the diagnosis because of the characteristic pattern of bone marrow edema. The diagnosis of BMES is a challenge for clinicians. Other causes of lower extremity pain, with poor prognosis, must be excluded. We present three cases of BMES. All three patients initially complained of mild lower extremity pain, which progressively deteriorated and led to a severe limitation of their daily activities. They were all treated conservatively by weight-bearing restriction and symptoms resulted within a few months. The aim of the present study is to outline this rare, benign pathology.

## Introduction

Bone marrow edema syndrome (BMES) is a syndrome characterized by bone pain and bone marrow edema on magnetic resonance (MRI). It is a distinct, benign disorder with a distinctive self-limiting course. Pain of the affected extremity and increased interstitial fluid in the bone marrow are the main aspects of the syndrome. It is a rare condition with an obscure etiology. There are different types of the syndrome published in the literature, such as transient bone marrow edema syndrome, transient osteoporosis of the hip and regional migrating osteoporosis [1]. 

Weight-bearing bones of the lower limbs are mainly involved, with a percentage as high as 98% [2]. The hip is the most frequently affected site of the syndrome with the bones around the knee, the ankle and the foot following with a sequence of decreasing frequency. In cases with migrating marrow edema, the joints are typically affected proximally to peripherally. The contralateral or more distal joints are usually involved several months later. Migrating patterns of the syndrome can present in 20% to 70% of the patients [3].

Laboratory findings are used to rule out other causes of bone marrow edema, such as malignancy and infectious diseases. Plain radiographs may be normal at the onset of the symptoms, but after three to six weeks they may expose demineralization of the bone. The joint space remains intact, and there are no apparent subchondral lesions [4]. MRI typically exposes a diffuse bone marrow edema with low signal intensity on T1-weighted images and high signal intensity on both T2-weighted images and short-tau inversion-recovery images [1].

We present three cases of BMES that have been isolated from our outpatient clinic. The aim of our study is to increase awareness of clinicians about this uncommon, reversible and benign disorder.

## Case presentation

Case 1

A 57-year-old male patient presented in the orthopedics outpatient clinic with a mild left hip pain. His symptoms had started two weeks ago. He had been working as a manual laborer, though there was no report of injury in the recent past. No known medical conditions or allergies were reported. X-ray imaging did not reveal any pathology, the patient was afebrile and there was no limitation of range of motion of the joint noted. Anti-inflammatory medication was prescribed, and restriction of heavy labor was advised.

**Figure 1:**
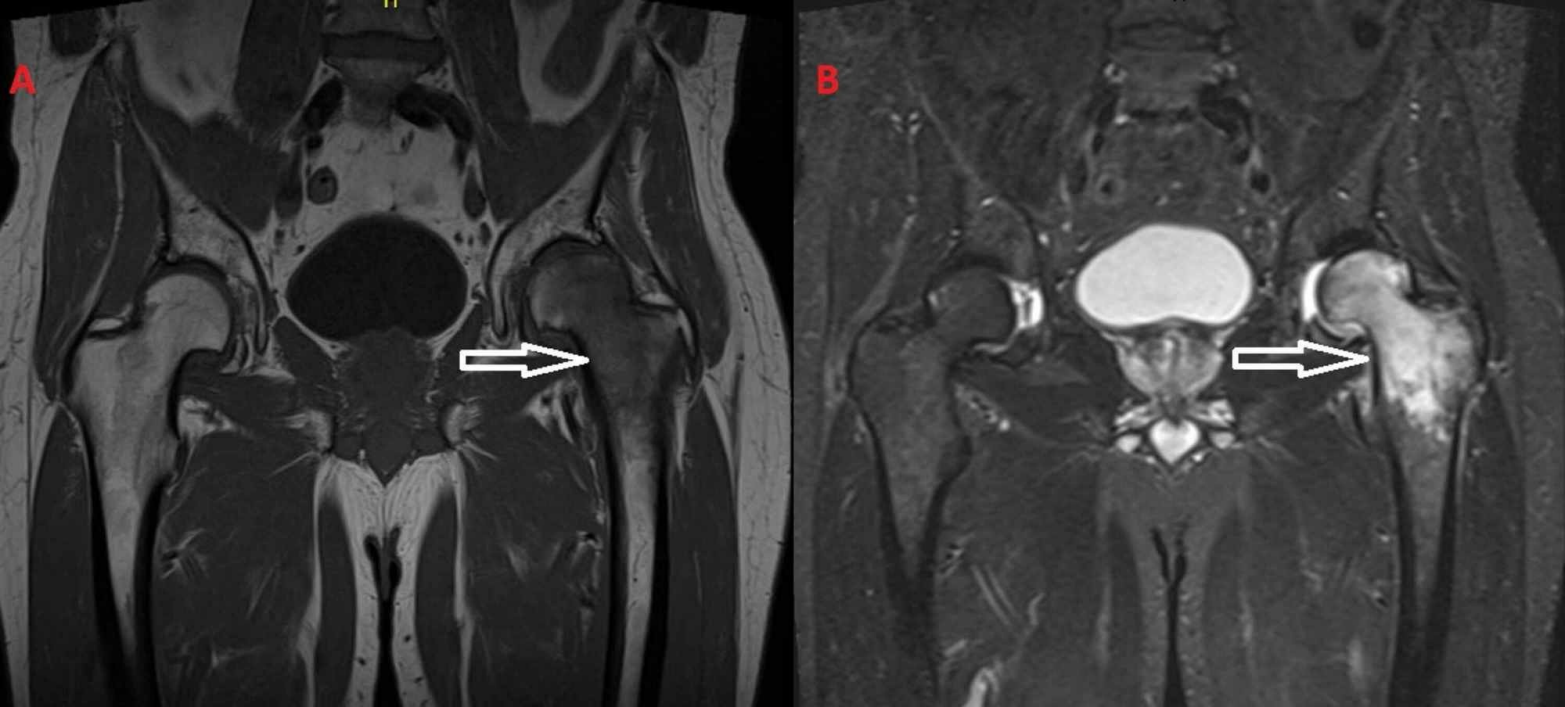
(A) T1-weighted images and (B) T2-weighted images from the MRI of the patient. The diffuse bone marrow edema of the left femoral head and the transtrochanteric region is apparent.

Ten days following his initial examination, in the follow-up control, the patient reported a progressive deterioration of the symptoms and disability to ambulate. A pelvic MRI was performed that revealed a diffuse bone marrow edema (Figure 1). Increased signal intensity was apparent in the T2-weighted images, and in contrast the signal intensity was decreased in the T1-weighted images. There was no evidence of subchondral fracture or disturbance of the joint space. The MRI images were suggestive of BMES. Laboratory tests that included inflammatory, tumor markers and bone profile were normal, excluding other pathologies. 

**Figure 2:**
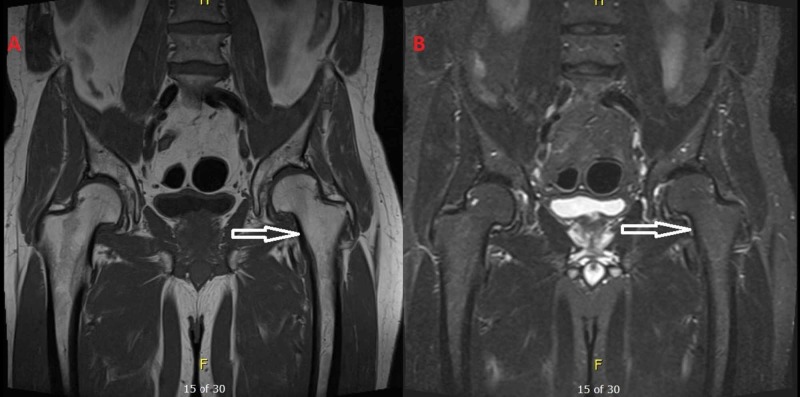
(A) T1-weighted images and (B) T2-weighted images from the MRI after the regression of the symptoms, five months after the initial diagnosis of bone marrow edema syndrome. The bone marrow edema has subsided.

Restriction of weight bearing was advised, and the patient’s condition was frequently clinically and radiologically evaluated. The iInitial underestimation of the progressive hip pain led to a four months of sick leave before recovering and returning to his work. Possible continuous microtrauma of the patient’s hip, because of manual labor, could have led to the development of the BMES and to the deterioration of the symptoms. Five months after the initiation of the symptoms, the patient was pain free, and returned to work and to his daily activities (Figure 2).

Case 2

A 55-year-old female patient was presented in our outpatient clinic with progressive right knee pain. Initial symptoms had appeared 10 days before examination. History of osteoporosis was mentioned, which was treated with zoledronic acid for the past two years. There were no known concomitant pathologies and no recent injury. History of heavy smoking in the past was mentioned, though a recent smoking cessation was outlined.

**Figure 3:**
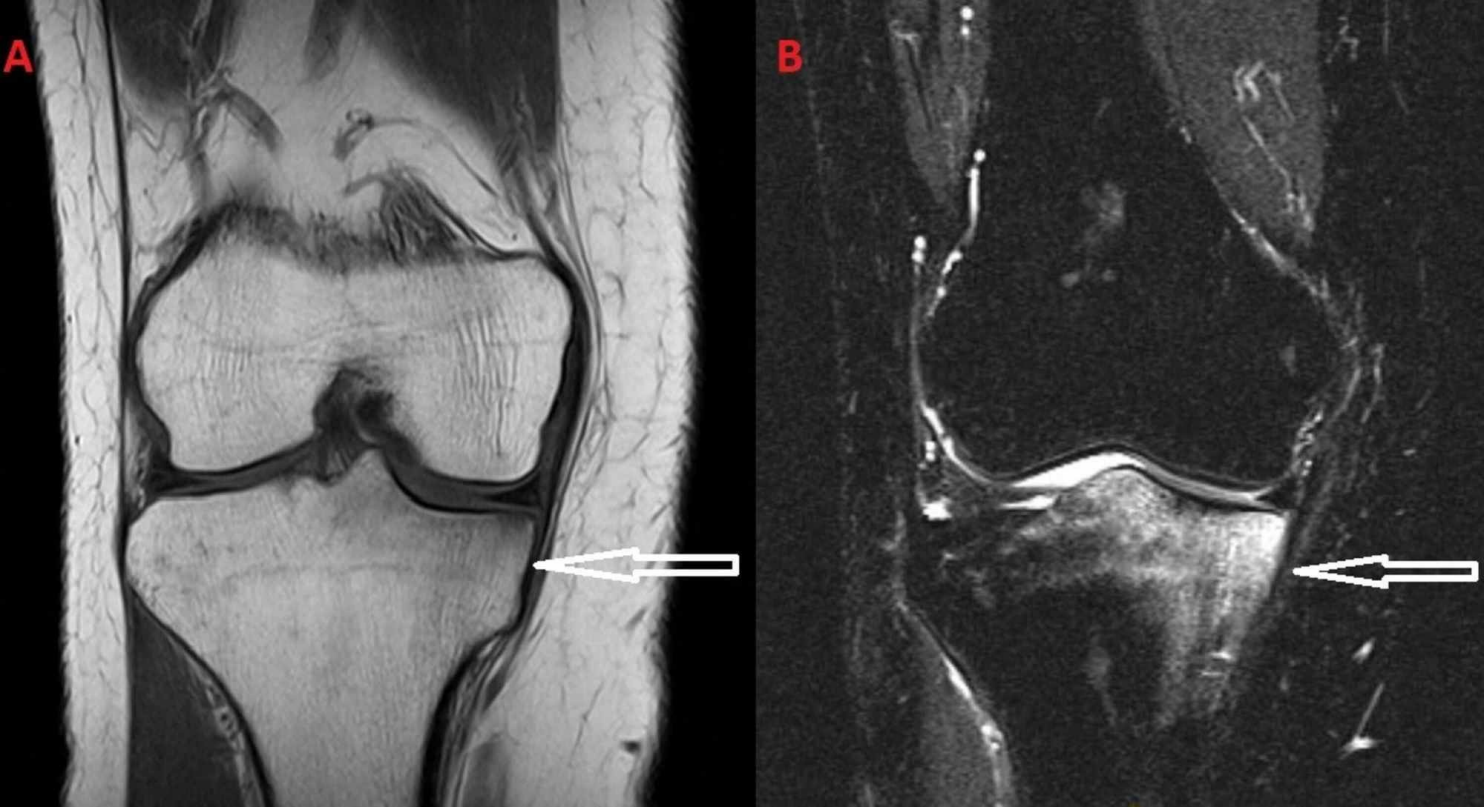
(A) T1-weighted images and (B) T2-weighted images from the initial MRI of the patient. The bone marrow edema is located at the medial tibial plateau.

The clinical examination revealed a full range of motion of the knee. The joint was not swollen, and no signs of redness or heat were noted. However, tenderness of the medial part of the proximal tibia was reported. X-rays of the affected knee were normal. Full laboratory tests were obtained, such as C-reactive protein, erythrocyte sedimentation rate, rheumatoid factor, human leukocyte antigen B27 and uric acid. They were evaluated to be within normal range. The MRI performed revealed a diffuse bone marrow edema of the medial tibial plateau (Figure 3). The characteristic of BMES presence of increased signal intensity on T2, along with decreased signal on T1-weighted images, was apparent.

**Figure 4:**
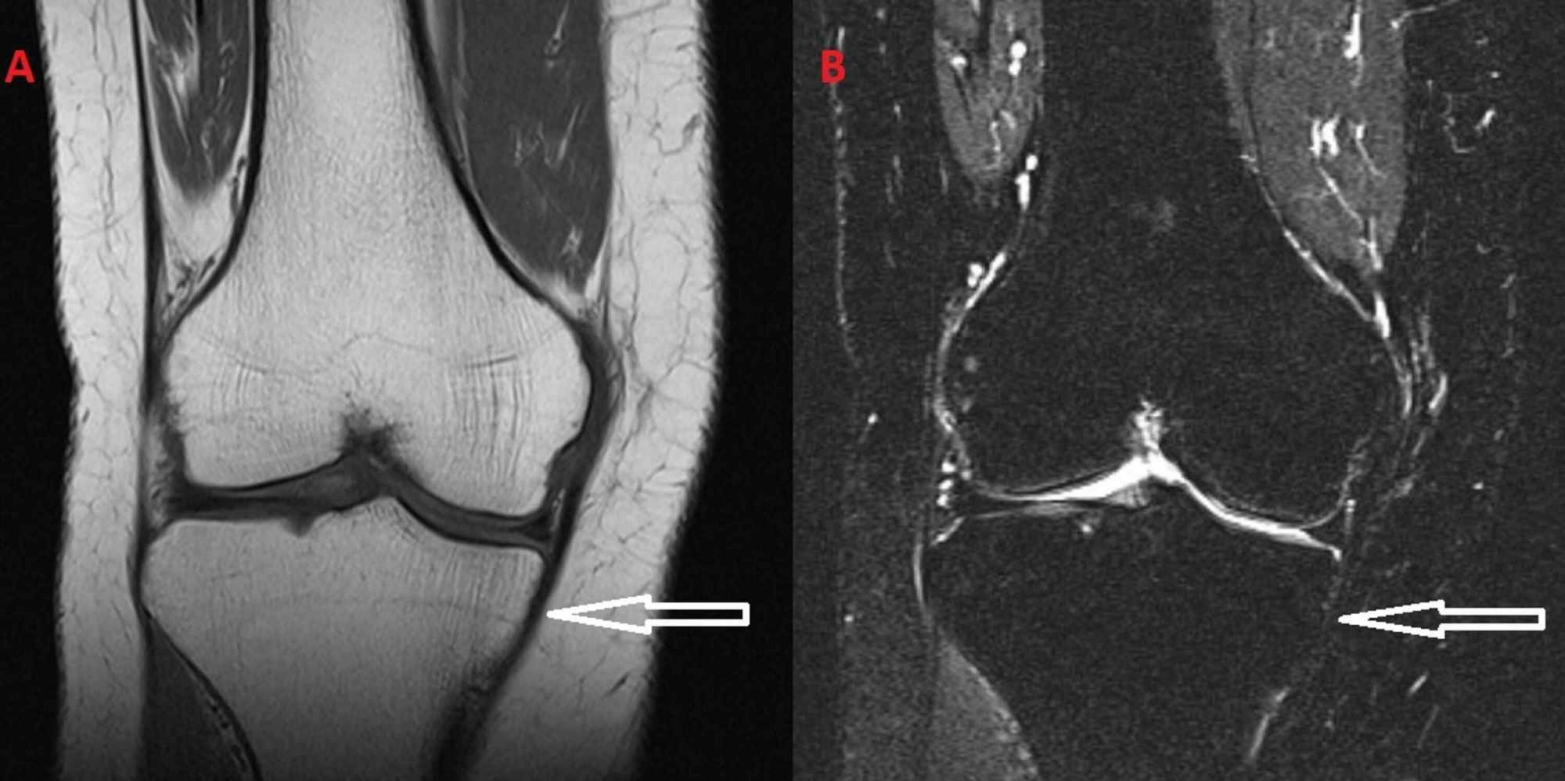
(A) T1-weighted images and (B) T2-weighted images from the MRI after the subsidence of the symptoms.

The patient presented with rapid deterioration of the symptoms within few weeks. In the follow-up examinations, inability to ambulate was reported. Restriction of weight bearing was strongly advised, and anti-inflammatory medication was prescribed. The pain resolved gradually. Four months after the initial examination, the subsequent MRI revealed a subsidence of the bone marrow edema (Figure 4).

Case 3

A 59-year-old female patient was referred to our outpatient clinic reporting continuous pain of the right hip. There was no history of trauma in the recent past. Hip pain started one week before our examination. The patient was afebrile. The range of motion of the hip was normal, and Patrick’s test was negative. Treatment with vitamin D and calcium supplements and amlodipine was noted because of a history of osteopenia and hypertension.

**Figure 5:**
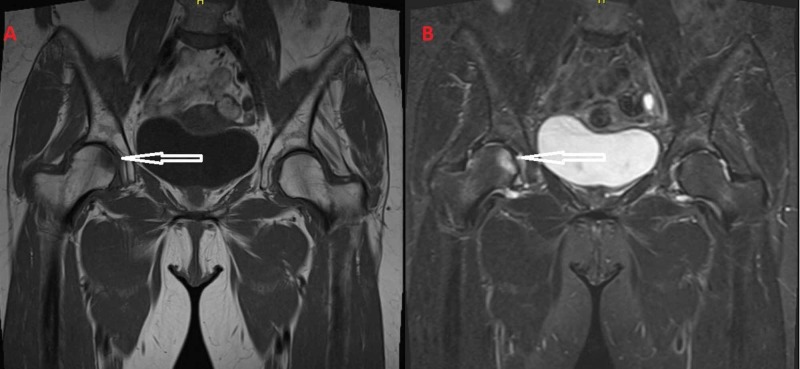
(A) Initial T1-weighted images and (B) initial T2-weighted images of the MRI of the right hip reveal the bone marrow edema of the right femoral head.

The x-ray did not reveal any evident pathology. An MRI was conducted that revealed a bone marrow edema located at the proximal part of the femoral head of the right hip (Figure 5). The joint space was normal, and there was no joint fluid accumulation. There was intense signal on T2-weighted images and decreased signal on T1-weighted images. Blood tests were normal, excluding other pathologies.

**Figure 6:**
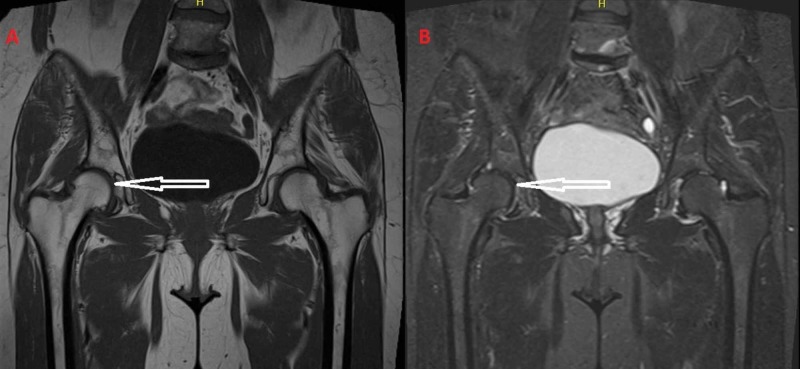
Regression of the bone marrow edema is evident at follow-up MRI (A: T1-weighted images and B: T2-weighted images).

Restriction of weight bearing was strongly advised. Simple painkillers, like paracetamol, were prescribed, as anti-inflammatory medication was not permitted because of the history of hypertension. At the follow-up examination, a rapid regression of the symptoms was evident. Three months after the onset of the pain and the MRI findings, the patient was asymptomatic and the new MRI revealed that the edema had resolved (Figure 6). 

## Discussion

Cases of transient osteoporosis of the hip were first reported in the literature by Curtiss and Kincaid in 1959 [5]. Duncan et al. in 1967 introduced the term regional migratory osteoporosis for migrating joint pain, affecting the weight-bearing joints, associated with regional osteoporosis [6]. In 1988, Wilson et al. first described MRI findings in patients with transient bone marrow edema regarding the hip and knee [7]. The term 'bone marrow edema syndrome' was introduced in the literature by Hofmann et al. in 2004 [8]. It is a distinct rare condition with unclear etiology. It commonly affects middle-aged men between 40 and 60 years old and women during the late trimester of their pregnancy. The onset of the symptoms is acute, including pain and stiffness. MRI is the examination of choice in these patients. Nonetheless, it is a diagnosis of exclusion.

Many theories have been proposed about the etiopathogenesis of BMES. Microvascular injuries, venous obstructions, abnormal mechanical stress, metabolic factors, endocrine factors and proximal nerve ischemia that can lead to denervation have been considered as possible causes of BMES. Furthermore, studies propose ischemia of bone, caused by transitory short-term intraosseous thrombosis, as the cause of BMES [4]. Trevisan et al. suggested regional accelerated phenomenon as the cause of BMES [9]. It has also been debated that BMES is an atraumatic form of reflex sympathetic dystrophy [10].

There are other conditions that can cause bone marrow edema. They can be classified according to their mechanism. The first group, which is associated with an ischemic mechanism, includes conditions such as osteonecrosis and osteochondritis dissecans. The second group of conditions, such as stress fractures, microfractures and bone bruise, has a mechanical etiology. In the third group that includes arthritis, tumor and postoperative edema, the bone marrow edema is reactive [11].

It is of utmost importance to exclude osteonecrosis from the differential diagnosis. Osteonecrosis can have similar symptoms in the early stages of the disease. Early diagnosis and aggressive treatment is important. The progress of osteonecrosis is irreversible. Similar bone marrow edema patterns are present in both conditions. Although osteonecrosis is not a rare condition, as nearly as 15000 new patients are diagnosed every year with this condition in the United States [12]. Findings in the MRI can help us distinguish the two conditions and avoid overtreatment of patients with BMES. Diffuse marrow edema, that can extend to the intertrochanteric area, with no focal or subchondral findings is suggestive of BMES. In osteonecrosis, band-like patterns on MRI are found before the appearance of bone marrow edema. The double-line sign on T2-weighted sequences is likewise diagnostic of osteonecrosis [13]. Differential diagnosis between the two conditions is imperative.

Some authors have suggested treatment of BMES with bisphosphonates, a prostacyclin analog (iloprost) and desonumab in order to accelerate the regression of the symptoms [14,15]. Moreover, there are some research studeis that suggest other treatment regimes, such as extracorporeal shock wave therapy and hyperbaric oxygen [16,17]. Nevertheless, these treatments are debatable because of the fact that BMES is a reversible condition, and it is not clear if the findings of these studies are attributable to the treatments.

## Conclusions

BMES is a rare condition with sudden onset of lower limb pain. The initially mild symptoms can lead to delay in diagnosis and treatment. It has obscure etiology and can rapidly progress from mild pain of the lower extremities to limitation of ambulation. Nonetheless, it is a benign and self-limiting disorder, which must be considered in the differential diagnosis of lower limb pain.
